# The complete mitochondrial genome of *Trachypenaeus curvirostris* (Stimpson, 1860)

**DOI:** 10.1080/23802359.2019.1660279

**Published:** 2019-09-06

**Authors:** Peng Zhu, Peng Luo, Pengliang Wang, Youhou Xu, Hong Zhang, Haiping Wu, Yongyan Liao, Fangmei Liang, Lili Liu

**Affiliations:** a Guangxi Key Laboratory of Beibu Gulf Marine Biodiversity Conservation, Beibu Gulf University, Qinzhou, Guangxi Autonomous Regions, China;; b Key Laboratory of Tropical Marine Bio-resources and Ecology (LMB), South China Sea Institute of Oceanology, Chinese Academy of Sciences, Guangzhou, Guangdong, China

**Keywords:** *Trachypenaeus curvirostris*, mitochondrial genome, Illumina sequencing

## Abstract

*Trachypenaeus curvirostris* is a common and important shrimp species in the shallow waters of Indo-West Pacific. In this study, the mitochondrial genome of *T. curvirostris* was determined for the first time using next-generation sequencing; the overall base components of mitogenome consisting of 15968 bp was 35.16% (5614 bp) for A, 33.51% (5351 bp) for T, 11.54% (1842 bp) for G, 19.80% (3161 bp) for C, and its GC content was 31.34%. The mitochondrial circular genome was composed of 13 protein-coding genes, 22 transfer RNAs, 1 D-loop and 2 ribosomal RNAs. Polygenetic analysis showed that the *T. curvirostris* was more closed to *Parapenaeopsis hardwickii*.

The cocktail shrimp *Trachypenaeus curvirostris* is an ecologically and economically important shrimp species in the shallow waters of Indo-West Pacific (Zhang 1983), with wide range of distribution from China and Australia to East Africa and the Red Sea, once contributing more than 50% of the total shrimp catch in China and Korea, with annual harvest of more than 300,000 t landed (Cha et al. 2004; Song et al. 2004). Comparing the historical density of resource, the resource of *T. curvirostris* in the East China declined greatly from 180.9 kg/km^2^ in the 1980s to 16.8 kg/km^2^ in 1990s (Han et al. 2015). The decline in species resource will decrease its genetic variation, which might afford an increasingly serious population status to the species. Despite its high commercial value and the urgent need to understand their stock structure, there is still no information available on its population genetics in the South China Sea. Hence, the complete mitochondrial genome sequence of *T. curvirostris* was sequenced, assembled and characterized, which could provide important genetic data for studying of population genetics, molecular phylogenetics and evolutionary relationship of *T. curvirostris*.

Total genomic DNA was isolated from each species using approximately of muscle tissue. Total DNA was eluted in sterile deionized water and was stored at −20 °C. The specimen was collected at China-Vietnam Common Fishery Area, Beibu Gulf, China (19.464 N, 107.391E) and stored at Herbarium of Ocean College in Beibu Gulf University (T.C.001). Paired-end library (450 bp) was sequenced using Illumina Hiseq4000 platform, with 150 bp pair-end sequencing method. The mitochondrial genome assembly using the chloroplast & mitochondrion assemble (CMA)V1.1.1 software (Guangzhou SCGene Co., Ltd, Guangzhou, China), which was based on sequencing reads’ overlap and paired-end relationship. Protein-coding genes and rRNA genes were annotated with blast+(2.5.0) with allied species, and tRNAs were predicted with tRNAscan-SE v2.0 (http://lowelab.ucsc.edu/tRNAscan-SE/) (Lowe and Chan 2016). 14,464 raw reads with average length of 150 bases and 2,169,600 nt were obtained, with average reads depth of 135.9X. Our research findings revealed that the circular genome is 15,968 bp, which consists of 13 protein-coding genes(PCGs), 1 D-loop, 2 rRNAs genes, 22 tRNAs genes, showing that the gene composition and arrangement are more closed to reported *Parapenaeopsis hardwickii* (Yang and Li 2018). The contents of A, T, G, and C in mitochondrial genome were 35.16, 33.51, 11.54, 19.80%, respectively. An overall GC content of whole mitochondrial genome is 31.34%. The sequence was deposited in GenBank (GenBank: KY021068). A phylogenetics analysis was conducted on 17 mitochondrial genomes. Maximum likelihood (ML) method was used to phylogenetic analysis. The best-fit models of evolution for the coding genes was selected by jmodeltest2 (https://github.com/ddarriba/jmodeltest2) (Darriba et al. 2012). RAxML v8.0.0 (RAxML Version 8 2014) was used to build the tree with 1000 bootstrap (Figure 1). Phylogenetic analyses showed that *T. curvirostris* was more close to *P. hardwickii* than *others.* The compete mitochondrial genome of *T. curvirostris* provides important genetic information for understanding phylogenetic relationships of crustacea mitochondrial genome.

**Figure 1.**
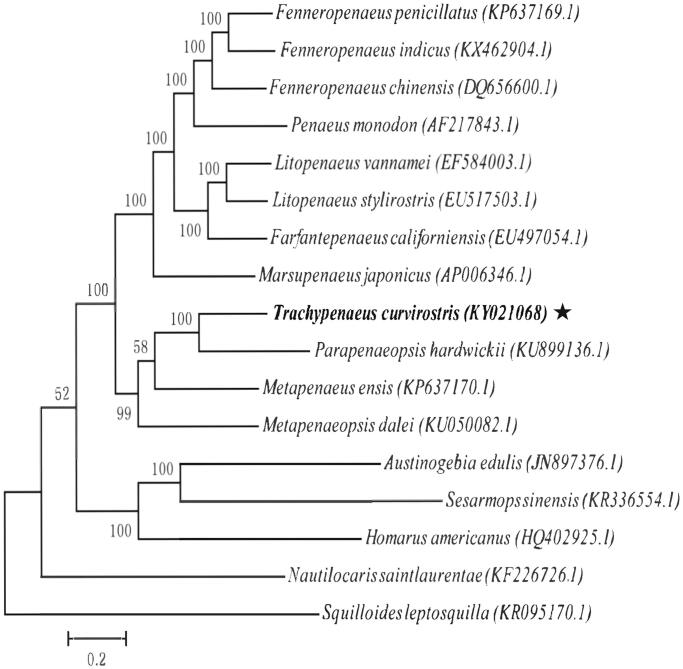
Phylogenetic relationships of Prosobranchia based on 17 mitochondrial genomes using NJ method. GenBank accession numbers: *Fenneropenaeus penicillatus* (KP637169.1), *Fenneropenaeus indicus* (KX462904.I), *Fenneropenaeus chinensis* (DQ656600.1), *Penaeus monodon* (AF217843.1), *Litopenaeus vannamei* (EF584003.1), *Litopenaeus stylirostris* (EU517503.1), *Fatfantepenaeus califorrniensis* (EU497054.1), *Marsupenaeus japonicus* (AP006346.1), *Truchypenueus curvirostris* (KY021068), *Parapenaeopsis hardwickii* (KU899136.1), *Metapenaeus ensis* (KP637170.l), *Metapenaeopsis dalei* (KU050082.l), *Austinogebia edulis* (JN897376.l), *Sesarmops sinensis* (KR336554.l), *Homarus americanus* (HQ402925.l), *Nautilocaris saintlaurentae* (KF226726.1), *Squilloides leplosquilla* (KR095170.1). Notes: The indicator (pentagram)means the complete mitochondrial genome of Trachypenaeus curvirostris, identified in this study.
